# Sugammadex-induced atropine-resistant bradycardia: clinical, pathophysiologic, and electrocardiographic considerations

**DOI:** 10.1186/s40981-020-00336-5

**Published:** 2020-05-07

**Authors:** Nicholas G. Kounis, Ioanna Koniari, George D. Soufras, Grigorios Tsigkas, Panagiotis Plotas, Periklis Davlouros, George Hahalis

**Affiliations:** 1 Department of Cardiology, University of Patras Medical School, Queen Olgas Square, 7 Aratou Street, 26221 Patras, Greece; 2 Electrophysiology and Device Department, University Hospital of South Manchester NHS Foundation Trust, Manchester, UK; 3 Department of Cardiology, “Saint Andrews” State General Hospital, Patras, Greece

**Keywords:** Anaphylaxis, Anesthesia, Kounis syndrome, Sugammadex

To the Editor:

In the very interesting report published in *JA Clinical Reports* [1], a 50-year-old woman without previous comorbidities, developed bradycardia and hypotension following intravenous sugammadex administration during anesthesia for transabdominal hysterectomy and right salpingo-oophorectomy. Propofol, fentanyl, remifentanyl, rocuronium, and levobupivacaine had been given preoperatively. Atropine was not effective but adrenaline recovered blood pressure and heart rate. Allergic skin signs were absent and tryptase and histamine were not measured. Electrocardiographic ST elevation in lead aVR and widespread ST depression were present. This report raises the following important issues:

## Perioperative medications

All above medications including sugammadex have been incriminated to induce anaphylaxis and Kounis syndrome [2–5]. Drugs can act as antigens inducing immunoglobin E (IgE) antibodies that are attached to the mast cell surface. Anaphylaxis ensues when antigens are bridged with their corresponding IgE antibodies and making at least 1000 bridges. IgE antibodies with different specificities can have additive effects and small, even sub-threshold numbers can join forces and trigger the cells to release their mediators [6, 7].

## Absence of skin manifestations in anaphylaxis

Tryptase or histamine was not measured due to absence of rash or itching. This had rendered the diagnosis of anaphylaxis difficult. Severe anaphylaxis and Kounis syndrome without skin involvement have been already reported [8, 9]. The bradycardia and hypotension following sugammadex could have been attributed to reduced cardiac output from leakage of plasma and volume loss. Volume loss reduces venous return and hampers or delays the release of mediators for reaching the skin areas and thus none applying their action [10].

## The “neglected aVR” lead

The patient’s electrocardiogram showed a unique sign of ST elevation in lead aVR, with reciprocal ST depression in the majority of other leads. These findings constitute new electrocardiographic manifestations of Kounis syndrome. The lead aVR, until recent years, was regarded as the “neglected lead” [11]. However, reports have shown that ST-segment elevation of more than 1.0 mm in lead aVR associated with widespread ST-segment depression in inferolateral leads, as in the described patient, best identifies severe left main or 3-vessel disease with 80% sensitivity and 93% specificity [12]. Urgent coronary angiography is necessary to confirm this and the diagnosis is high-risk non-ST segment elevation acute coronary syndrome that requires urgent revascularization and medical treatment that includes anti-platelets, aspirin, and heparin [13]. However, the same electrocardiographic findings can be present in type A dissecting aneurysm affecting the ascending aorta that expands and presses the left main artery and the coronary ostia. Whereas clinical picture is of acute myocardial infarction, the treatment is completely different and includes emergency surgery and avoidance of anti-platelets, aspirin, and heparin [14]. Such dilemma is easily solved by trans-thoracic echocardiography. The described patient was obese but had normal preoperative 12-lead electrocardiogram and past history free of comorbidities. In view of her perioperative electrocardiographic changes and the suspicion of type I Kounis syndrome angiographic evaluation postoperatively would have been helpful.

All above show that Kounis syndrome is a condition with variety of etiology, clinical, and electrocardiographic manifestations. During their everyday practice, anesthetists and surgeons should be always brought it in mind.

## Data Availability

Not applicable
